# Surgery

**Published:** 1877-10

**Authors:** 


### IV. Surgery.

About Shock, &c.—Nussbaum.—(*Bair. Ærztl. Intelligenz-Blatt*, 1877, No. 11, p. 107.) Many hitherto mysterious cases of collapse after operations can find a satisfactory explanation in one or the other of the following accidents:

(1.) *Acute Sepsis.* The application of septic fluids to a large and rapidly absorbing surface. Ovariotomy and various experiments prove this.

(2.) *Acute Anœmia.* After great loss of blood, particularly in previously anæmic persons and old people.

(3.) *Fat Embolism.* Particularly of the capillaries of the lungs after extreme comminuted fractures. Death often occurs in very robust individuals after this accident. It is ushered in with great dyspnœa. In all operations in the abdominal cavity, great danger is to be anticipated from rapid cooling of the peritoneum.

To prevent acute sepsis, we must employ sufficient antiseptic measures. By warmth and great care in our operations, we must prevent any great decrease of temperature in the peritoneum. As an illustration of what he means, Nussbaum relates a case of internal strangulation of the intestines. The operating-room, the antiseptic solution, and all cloths used, were made warm. The patient was wrapped up in warmed clothes. The strangulation was removed with the least possible exposure of the intestines. The man recovered rapidly and well.

Nussbaum thinks that by using the above precautions, and what we have already learnt of abdominal operations, it would be safe to operate from the abdominal cavity for radical cure of hernia.

